# 10,000-Times Diluted Doses of ACCase-Inhibiting Herbicides Can Permanently Change the Metabolomic Fingerprint of Susceptible *Avena fatua* L. Plants

**DOI:** 10.3390/plants8100368

**Published:** 2019-09-24

**Authors:** J António Tafoya-Razo, Ernesto Oregel-Zamudio, Sabina Velázquez-Márquez, Jesús R. Torres-García

**Affiliations:** 1 Departamento de Parasitología Agrícola, Universidad Autónoma Chapingo, Texcoco 56230, Mexico; jtafoyar@chapingo.mx; 2 Laboratorio de Ecología y Evolución Molecular, Centro Interdisciplinario de Investigación para el Desarrollo Integral Regional (CIIDIR) del Instituto Politécnico Nacional, Unidad Michoacán, Jiquilpan 59510, Mexico; eoregel@ipn.mx; 3 Laboratorio de Genética Ecológica y Evolución, Departamento de Ecología Evolutiva, Instituto de Ecología, Universidad Nacional Autónoma de México, Ciudad de México 04510, Mexico; svelazquez@ecologia.unam.mx; 4 Cátedras CONACyT, Ciudad de México 04500, Mexico

**Keywords:** non-target metabolomics, GC-MS, non-target site resistance, priming, hormesis

## Abstract

Intentional use of low dosage of herbicides has been considered the cause of non-target resistance in weeds. However, herbicide drift could be a source of low dosage that could be detected by weeds and change their metabolism. Furthermore, the minimum dose that a plant can detect in the environment is unknown, and it is unclear whether low doses could modify the response of weeds when they are first exposed to herbicides (priming effects). In this study, we determined the metabolomic fingerprinting using GC-MS of susceptible *Avena fatua* L. plants exposed to a gradient of doses (1, 0.1, 0.001, 0.0001, and 0x) relative to the recommended dose of clodinafop-propargyl. Additionally, we evaluated the primed plants when they received a second herbicide application. The results showed that even a 10,000-fold dilution of the recommended dose could induce a significant change in the plants’ metabolism and that this change is permanent over the biological cycle. There was no evidence that priming increased its resistance level. However, hormesis increased biomass accumulation and survival in *A. fatua* plants. Better application methods which prevent herbicide drift should be developed in order to avoid contact with weeds that grow around the crop fields.

## 1. Introduction

Evolution of herbicide resistance is the result of the strong selective pressure exerted by herbicides on weed populations [1]. This selection pressure is so strong that Harper predicted the evolution of herbicide-resistant weed populations even before the appearance of the first report [2]. Early studies of resistant populations attributed resistance to non-synonymous mutations in specific domains in herbicide target genes (Target-Site Resistance, TSR). These mutations change the protein conformation and limit herbicide effectiveness [1,3]. For this reason, some weed management models suggested the reduction of herbicide dosage to reduce the selection pressure in weed populations [4], a recommendation which was implemented in many countries. However, this practice had undesired effects [5], including an increase in the number of populations with metabolic adaptations to degrade herbicides (Non-Target site Resistance, NTSR) [6]. This type of resistance is due to the increase in the activity of specific enzymes such as Cytochrome oxidase P450 (hereafter P450) and Glutathione-S transferase [6]. NTSR has increased over time and has the potential to become a severe problem due to the capacity of degradation of multiple herbicides [1]. Moreover, experiments have shown that this type of resistance could evolve in less time than genetic resistance [7,8,9].

Other documented effects of low herbicide dosage on weeds are hormesis and priming [10,11,12]. Priming is defined as a physiological state caused when plants exposed to a low dose of a stressful agent (bacterial, fungus, herbicides, among others) develop an adaptative response, which increases their resistance to subsequent exposure [13]. This phenomenon has been documented in tumor cells, and it is possible that it is a mechanism of NTSR [14]. On the other hand, hormesis is growth stimulation at low doses of herbicides [12]. This phenomenon has been reported in many herbicide modes of action [15,16,17], and has even been proposed to use the hormetic effects to increase yield in some crops [18,19].

Due to the rapid evolution of NTSR based in P450, the current weed management practices suggest avoiding the application of herbicides at lower-than-recommended doses to prevent the risk of development of metabolic resistance [1,5,6]. However, several factors lead to this continuing in practice. On the one hand, farmers frequently reduce the dosage of herbicide to try to save money by purchasing less herbicide (Torres–Garcia, personal observation). Another cause of low herbicide dosage is late application; herbicide use is recommended when weeds are about 10 cm tall. However, farmers often apply chemical control when weeds are many times higher than the recommended size, thus diluting the effective dose (A Tafoya, Personal Observation).

Even when following all recommendations for their use, from manual application to airplane spraying, herbicides can be spread unintentionally, for example by leaf contact between treated and untreated plants, protection by taller plants, and drift of spray particles to nearby fields [20]. Clouds of vapor carrying nano-drops of herbicide could modify the metabolism of surrounding weeds. In highly advanced agricultural systems, this is not a problem, but only a small proportion of global agriculture has this level of mechanization.

With respect to herbicide spray particle drift, some basic questions that we asked are: (1) What is the minimum dose of herbicide that a plant could perceive? (2) Do weeds that received a non-lethal dose show differences in metabolism and survival when they receive a second application (i.e., are there priming effects)? and (3) Are the metabolic changes transgenerational, and therefore, a possible cause of metabolic resistance? The last question has been well responded by Neve and Powles [8]. However, the first two questions remain unclear. In this study, we are interested in generating information that could respond to the two initial questions.

Plant metabolism is complex, with a large number of chemical compounds and interactions among them, making the identification and quantification of all metabolic changes a complicated job [21,22,23]. The use of high-throughput metabolic methods can detect minimal changes in the metabolic state, giving a fingerprint of the metabolic state of the plant [24]. The metabolome is the final result of the plant’s response and could provide us with a detailed “snapshot“ of the changes caused by herbicides [25,26]. In this study, we applied a non-target metabolomic scope based on the identification of punctual metabolomic fingerprinting using GC-MS as an analytical approach. We expected that if plants could detect the presence of herbicides in the environment, then they would show changes in their global fingerprint compared to untreated plants, and this fingerprint would be different in plants that had previously been exposed to herbicides than those that had not been previously treated.

## 2. Results

### 2.1. Experiment 1. Biomass Accumulation and Survival

**Figure 1:**
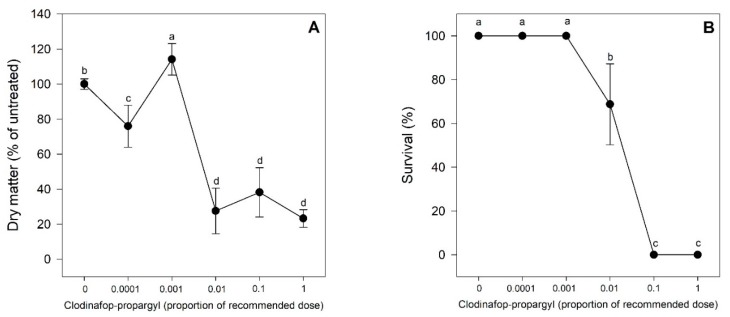
Dry matter accumulation (**A**) and survival (**B**) of susceptible *Avena fatua* L. plants treated with clodinafop-propargyl in proportional doses of the recommend rate (1x, 0.1x, 0.01x, 0.001x, 0.0001x, and 0x). Statistical significances are indicated with different letters. Datapoints represent mean values, and vertical bars represent standard errors. When they are absent, they are smaller than the symbol (*n* = 4).

The application of clodinafop-propargyl showed a significant reduction in the dry matter accumulation of the studied biotype compared to unexposed plants, except at the 0.001x dose. Plants grew and accumulated significantly more dry matter even in the lowest dose (0.0001x the recommended dose). In plants treated with 0.001x, there was an increase in the dry matter accumulation caused by hormesis (Figure 1A). Dry matter accumulation showed a drastic reduction at the doses from 0.01x to 1x. Survival, on the other hand, was not affected in the two least concentrated doses (0.0001x and 0.001x) of clodinafop-propargyl, but there was a significant decrease in survival at the 0.01x dose (63% survival), and there was 100% mortality at both the 0.1x and 1x doses (Figure 1B).

#### Metabolomic Fingerprinting of the First Experiment

**Figure 2:**
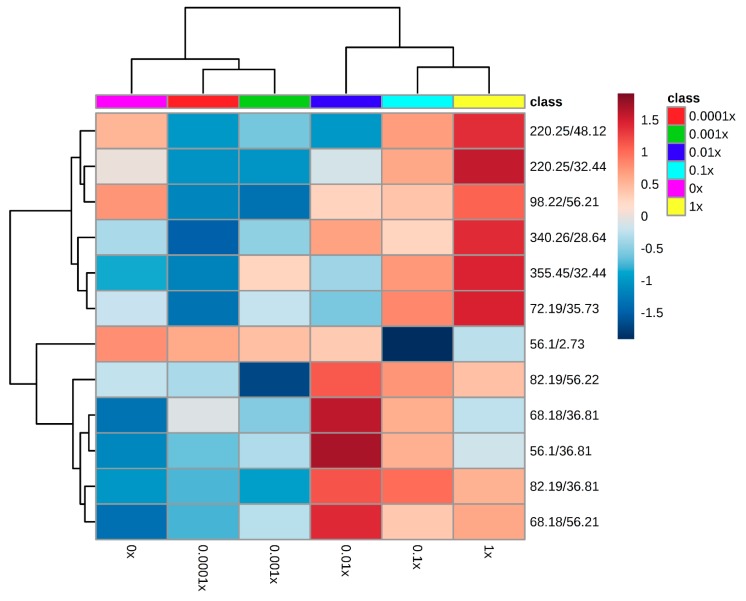
Metabolomic fingerprint of susceptible *Avena fatua* L. plants (*n* = 4) at 24 h after been sprayed with clodinafop-propargyl in proportional doses of the recommend rate (1x, 0.1x, 0.01x, 0.001x, 0.0001x, and 0x). The heatmap was constructed with the 12 metabolites that showed *p*- and *q*- values ≤ 0.05. Colors represent the abundance of metabolites; the blue color indicates down-expression and red color over-expression. The metabolites are clustered according to their Pearson correlation as a distance function, and the Ward clustering algorithm, the significance of the branches were of *p* ≤ 0.05.

The metabolic fingerprint of the susceptible biotype of *A. fatua* with the application of clodinafop-propargyl was obtained by GC-MS recording a total of 67 metabolites. For the construction of the heatmap, we used only the 12 metabolites that had *p*- and *q*- values ≤ 0.05. The resulting heatmap shows significant changes in the metabolism caused by herbicide application, even with the most diluted concentration sprayed (Figure 2).

The dendrogram along the top Figure 2 shows the grouping among treatments. In this dendrogram, we found the formation of two main groups (branches). One of these branches (left side of the heatmap) includes the lower doses (including the control treatment; 0x, 0.0001x, and 0.001x; Figure 2) This grouping also corresponds with that observed in the dry matter accumulation and survival. Inside this branch, the control treatment (0x) comprised a different subgroup. This indicates that plants showed changes in the metabolic fingerprint even at a 10,000-fold reduction of the recommended herbicide dose. In the case of treatment with the 0.001x dose, there was an apparent hormetic effect on dry matter accumulation, but there was no evidence of significant changes in their expression pattern that would explain this effect, and the heatmap did not reveal metabolic differences between the 0.0001x and 0.001x treatments. The second main group (right side of the heat map) included the higher-dose treatments which caused the highest dry matter reduction and mortality. In this branch, the 0.01x treatment was divided as a subgroup from the 0.1x and 1x treatments.

The dendrogram along the left side of the heatmap reveals the relationship among the metabolites detected. This dendrogram also has two main branches; in the upper section, there was a marked difference in the expression of the metabolites between the 0.0001x and 1x treatments. Metabolites of the 0.0001x treatment are shown in shades of blue, indicating that those metabolites were down-expressed. In contrast, the 1x treatment showed an over-expression of the same metabolites.

The second branch of the dendrogram (the lower half of the heatmap), according to random forest analysis, contains the five most important metabolites for classification (82.19/36.81; 68.18/36.81; 68.18/56.21; 56.1/36.81), such metabolites correspond to Hexane-2,6-di(isonitrile), 1-(formyloxymethyl)-Z-3, 17-Octadecadien-1-ol, and acetate(S)-2-methylbutanoic acid methyl ester 2-methylpropanoic.

In this zone, there was an evident change in expression. Treatments with the lowest doses (including 0x) showed a down-expression of those metabolites, while treatments with the most concentrated doses and with more biological changes (several reductions in the dry matter and high mortality) show overexpression of those metabolites.

### 2.2. Experiment 2. Biomass Accumulation and Survival

**Figure 3:**
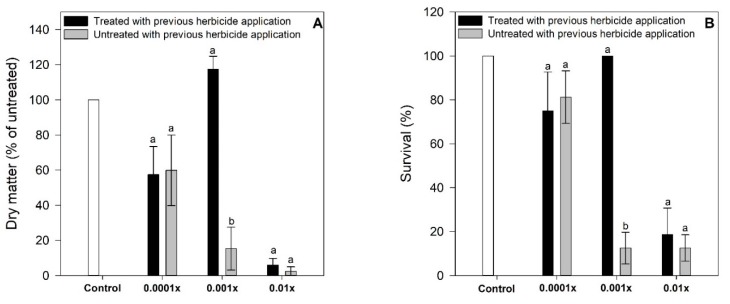
Dry matter accumulation (**A**) and survival (**B**) of susceptible *Avena fatua* L. plants that have been treated previously, and plants that received their first application of clodinafop propargyl at rates of 0.01, 0.001, 0.0001x of the recommended dose. Control plants only were sprayed with distillate water and adjuvant. Statistical significances are indicated with different letters. Datapoints represent mean values, and vertical bars represent standard errors. When they are absent, they are smaller than the symbol (*n* = 4).

In the plants treated with 0.0001x and 0.01x doses, we did not find differences in the growth and survival among plants that had been previously sprayed with herbicides versus plants that were receiving their first application. In the case of the 0.001x treatment without previous herbicide application (0.001x-U), there was a significant increase in dry matter and survival (Figure 3). A significant increase of the dry matter was observed, even respect to control (60% of the rise). The survival was of the 100% in all pots sampled.

#### Metabolomic Fingerprint the Second Experiment

In the second experiment, 51 metabolites were detected with *q*-values ≤ 0.05, and 46 metabolites had significant *p*-value ≤ 0.05, so these 46 were used to construct the heatmap. Four of those metabolites were shared with the first experiment: 220.25/48.11, 67.15/36.81, 72.12/35.72, and 220.25/32.45. According to the NIST (National Institute of Standards and Technology, Gaithersburg, MD, USA) library, such metabolites were 2-Methylamino-3-methylbutanoic acid, 2-methylpropanoic acid, Benzoic acid methyl ester, and 1-(p-Methoxycarbonylphenyl)-5-phenyl-3-(2-pyridyl)-2-pyrazoline, respectively.

**Figure 4:**
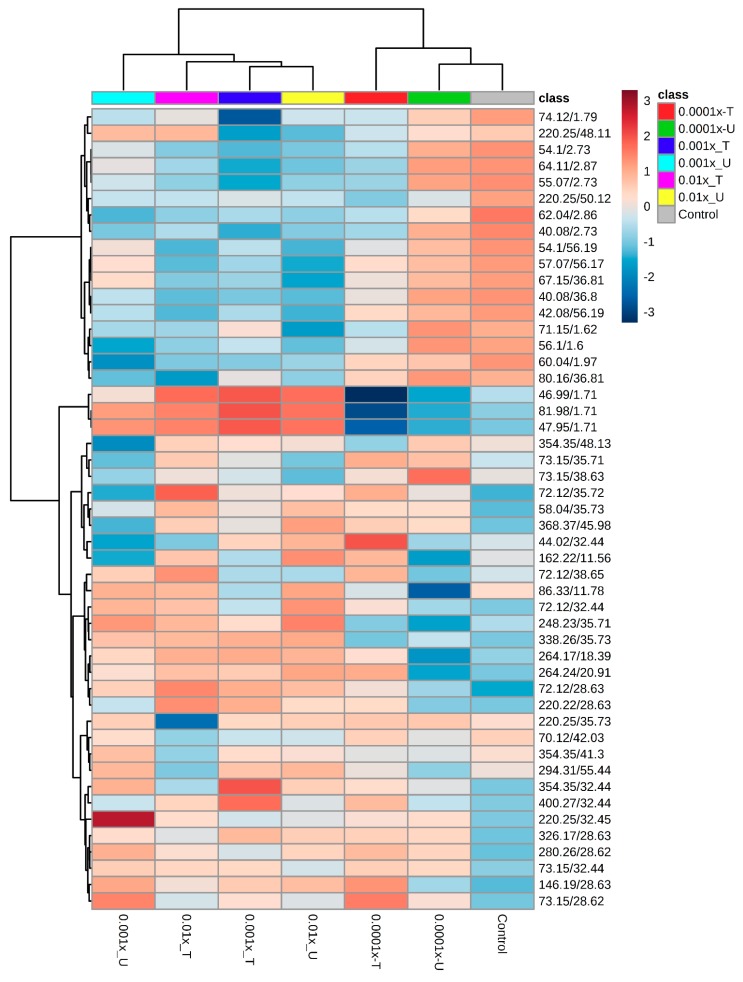
Metabolic fingerprint of susceptible *Avena fatua* L. plants that have been treated previously (T = Treated) and plants that received their first application (U = Untreated) of clodinafop propargyl at rates of 0.01, 0.001, and 0.0001x of the recommended dose. The heatmap was constructed with the 46 metabolites that showed *p*- and *q*- values ≤ 0.05. Colors represent the abundance of metabolites; the blue color indicates down-expression and red color over-expression. The metabolites are clustered according to their Pearson correlation as a distance function, and the Ward clustering algorithm, the significance of the branches were *p* ≤ 0.05.

The resulting heatmap shows that treatments were grouped into two main branches (Figure 4). One branch (Figure 4; right side of the heatmap) was conformed of the control, and the two most dilute herbicide applications (0.0001x-U, 0.0001x-T). Within this branch, the control and 0.0001x-U treatments had a very similar fingerprint, and for this reason, were grouped into the same sub-group. On the other main branch, all treatments were grouped in closed sub-branches. The only treatment that showed differentiation in this sub-branch was the treatment 0.001x-U. This same treatment also showed significant differences in dry matter and survival.

The dendrogram along the left side of the heatmap in Figure 4 shows the marked differences in metabolite expression of the treatments. The upper half of the heatmap shows that treatments with the lowest dose of herbicide (including the control) had increased expression of 17 metabolites. On the other branch, those metabolites were expressed less. In the lower branch, a set of tree metabolites had an inverse expression pattern compared to the other treatments. The metabolites 46.99/1.71, 81.98/1.71 and 47.95/1.71 (2-Nonen-1-ol, (S)-2-methylbutanoic acid methyl ester, and 3-Buten-1-ol, 2-methyl, respectively) were expressed less in the treatments with low doses of herbicide, while in the higher dose treatments, they were expressed more.

The 0.0001x-U treatment was grouped into a separate group in the heatmap, and it also had a significant change in dry matter and survival. In the fingerprinting of this treatment, a marked down-expression of eight metabolites (354.35/48.13, 73.15/35.71, 73.15/38.63, 72.12/35.72, 58.04/35.73, 368.37/45.98, 44.02/32.44, and 162.22/11.56) constitutes a notable difference compared to all of the other treatments. These compounds correspond to aromatic compounds as 1, 2-Hexadecanediol, trans 3-penten-1-ol, 3-methyl-2-buten-1-ol, Benzoic acid methyl ester, 1-pentenal, 1-(p-Methoxycarbonylphenyl)-5-phenyl-3-(2-pyridyl)-2-pyrazoline, and 2-heptanol. All treatments that received herbicide application showed increased expression of these metabolites; while the control treatment also showed decreased expression of the same metabolites, but this expression is not so evident as in treatment 0.0001x-U.

## 3. Discussion

In this study, we simulated the effect of herbicide drift (or any event that leads to exposure to a low herbicide dosage) on the metabolism of weeds surrounding crop fields. Our principal objectives were to determine: (1) What is the minimum dose of herbicide that a plant can perceive, and (2) if plants that received non-lethal doses show differences in their metabolism, growth, and survival when receiving a second application. The results showed that susceptible plants of *A. fatua* could detect the presence of clodinafop-propargyl in doses diluted by 10,000 fold with respect to the recommended dose. The changes observed in the metabolomic fingerprint were present at least 21 days after herbicide application. When the plants received a second application of herbicide, they showed differences in the metabolomic fingerprint compared to plants that were not treated previously, but this did not lead to changes in biomass accumulation and survival. Hormesis was observed with the application of a dose of 0.001x, but this did not increase their tolerance to a second application. In the second experiment, these hormetic effects also increased survival; this means that changes in plant metabolism can occur at extremely low doses and have effects on the plants’ fitness, depending on plant age.

*A. fatua* plants showed reductions in their dry matter accumulation even at low doses (10,000 fold dilution of the recommended dose). This dry matter reduction was caused by the high effectiveness of herbicide in susceptible plants [27]. However, it is possible that other susceptible biotypes could display a different degree of susceptibility [28]. The metabolomic fingerprinting observed in the first experiment was congruent with the observations in dry matter and survival. Metabolic changes were observed 24 h after the herbicide application, with clear differentiation in the expression of some metabolites among treatments that showed high levels of damage and mortality. These differences in the fingerprint could be used as a predictive tool for determining susceptibility or resistance. Torres-García et al. [26] using Direct-Injection electrospray ionization mass spectrometry (DIESI) detected differences in the fingerprints of multiple herbicide-resistant biotypes of *A. fatua* (resistant to ACCase- and ALS-inhibiting herbicides) when sprayed with herbicides of each mode of action.

The analytical approach used in this study (GC-MS), has the disadvantage that only volatile compounds that can be detected, compared with other methods such as UPLC-MS, EI-MS, among others [29,30]. However, the 12 metabolites in the first experiment and 46 in the second fulfilled the requirements of *q*- and *p*- values ≤ 0.05, confirming their participation in the metabolic response to herbicide application. The non-target metabolic approach used also has the disadvantage that the identification of metabolites could be spurious due to the lack of standards for each metabolite found. However, one of the objectives of this study was to determine the minimum dose of herbicide that a plant can perceive, and it was accomplished. Besides, this could be an initial step for accurately determining the metabolites that participate in the plants’ response to herbicides, i.e., the four metabolites that were shared in the two experiments and putatively were identified as 2-Methylamino-3-methylbutanoic acid, 2-methylpropanoic acid, Benzoic acid methyl ester, and 1-(p-Methoxycarbonylphenyl)-5-phenyl-3-(2-pyridyl)-2-pyrazoline.

The second experiment demonstrated that previous herbicide application (priming) did not have any effect on the biomass and survival during a second herbicide application. The expectation is that primed plants develop defense responses that are faster, stronger, and more sustained than in plants that were not primed [10]. However, in the phenotypic traits measured, this did not occur. On the other hand, there were significant changes in the metabolomic fingerprint. These changes caused by stimulation by low doses of herbicides were present throughout the biological cycle. The metabolomic fingerprint of plants that received a prior dose of herbicide was different from those that were receiving herbicide application for the first time. These effects have been called “metabolic memory” or “priming” and it has been demonstrated that this effect can be passed to the next generation, indicating an epigenetic component of transgenerational inheritance [11]. These transgenerational priming effects are likely one of the factors on the evolution of NTSR based in the overexpression of P450 genes. Neve and Powles [8,9] reported that recurrent selection at low doses for three generations caused a 55-fold increase in the resistance index. Later, Yu et al. [31] confirmed that the resistance mechanism of these biotypes was based on the over-expression of P450.

An exception to priming effects on metabolomic fingerprinting was the treatment 0.0001x-U. This treatment was grouped in the same branch as the control treatment. In this case, it is possible that the dose of 0.0001x (10, 000 diluted) was too low to provoke a metabolic change in plants with larger sizes (around 45 days after seedling). This elevated sensitivity is a factor to consider since the metabolic response of weeds to herbicide drift will depend on the plant size.

The treatment that showed hormetic effects was the only one that increased in survival. In addition, its metabolic fingerprint differed from those of the other herbicide treatments, particularly in eight metabolites (1, 2-Hexadecanediol, trans 3-penten-1-ol, 3-methyl-2-buten-1-ol, Benzoic acid methyl ester, 1-pentenal, 1-(p-Methoxycarbonylphenyl)-5-phenyl-3-(2-pyridyl)-2-pyrazoline, and 2-heptanol). However, the hormesis is a complex phenomenon that cannot be explained by the expression of only eight metabolites, and more research is needed [12].

The results presented in this study demonstrated the high sensitivity of susceptible biotypes to the presence of herbicides (clodinafop-propargyl) in the environment. This could have implications in the contamination caused by the application methods used. The unintentional low dosage caused by the drift of micro drops carrying ultra-diluted doses of herbicide can be detected by susceptible plants that grow around the crop fields and may cause metabolic resistance. Improvement in application methods is required to avoid drift. This study also documents the lack of priming effects in response to herbicides in *A. fatua* and that there is an increase in size and survival caused by hormesis.

## 4. Methods

### 4.1. Study System

*Avena fatua* L. is considered as the world’s second-worst herbicide-resistant weed due to their worldwide presence in cereal-growing regions [32]. This weed has evolved resistance to at least seven modes of action (antimicrotubule mitotic disrupter and ACCase-, ALS-, PPO-, cell elongation-, long-chain fatty acids-, and lipid- inhibitors). A single biotype from Canada has even evolved multiple resistance to 5 modes of action (ACCase-, ALS-, PPO-, long-chain fatty acids-, and lipid- inhibitors) [33]. In recent years, the increase of cases of metabolic resistance also documented *A. fatua* biotypes [6].

The *A. fatua* biotype used in this study was collected in an alfalfa crop, in a zone where cereal has not been produced for the past 10 years (A Tafoya, personal observation). The susceptibility was confirmed in greenhouse conditions, and this biotype has been used in other studies as a susceptible biotype [26,34].

### 4.2. Experiment 1. Determination of the Minimum Dose of Herbicide That Produces Changes to Plants’ Metabolism

We separated this study into two experiments to address each of the following questions separately: (1) What is the minimum dose that causes changes in the plants’ metabolism? and (2) do weeds that received a non-lethal dose differ in metabolism and survival upon receiving a second dose compared to plants that are receiving their first dose (i.e., are there priming effects)?

Around 500 caryopses of the collected susceptible biotype of *A. fatua* of similar size and weight were selected. This selection was made to ensure the physiological maturity of the caryopses used. The florets (lemma and palea) were removed manually to synchronize the germination. Then, the caryopses were disinfected by immersing them in a solution of water and sodium hypochlorite at 5% for 10 min and washed three times with distilled water. Disinfected caryopses were placed in Petri dishes with wet filter-paper and maintained to 20 °C. Germination was considered to have occurred when the radicle measured 3 mm in length.

Plastic pots with 500 mL capacity were filled with a mix of peat moss and agrolite in a proportion of 1:1. In each pot, six seedlings were planted. Pots were maintained in a growth chamber with a light flux of 440 µmoles m^−2^ s^−1^, photoperiod of 16 h light/8 h dark, and constant temperature of 18 °C. The substrate was maintained near field capacity during the experiment. The plants were fertilized with Steiner nutrient solution (1x) every 14 days (50 mL per pot).

To determine the minimum dose of herbicide that a plant can detect in the environment, we used the recommended rate of clodinafop-propargyl (60 g a. i. ha-1, 1x) and four 10-fold consecutive dilutions (0.1x, 0.01x, 0.001x and 0.0001x). Each dilution represents a treatment, and for the preparation of each dilution, the adjuvant concentration was the same. The control conditions consisted of the application of distilled water and adjuvant (0x). This spectrum of doses ranges from the recommended dose to an extremely diluted dose 10, 000 fold weaker than the recommended dose, which could represent the spray particle drift that occurs during herbicide application.

The herbicide treatments were applied when plants were at least 10 cm tall, using a pressurized CO_2_ plot sprayer calibrated to a spray volume of 200 L per hectare. Four replicates were carried out for each dose. After the application of herbicides, pots were kept separate for 6 h to ensure the penetration of herbicide and avoid contamination among treatments.

Plant tissue was sampled for metabolomic analyses 24 h after the herbicide application; at that time, plants did not show damage symptoms in any of the treatments. Two plants per pot were taken, leaving four individuals in each pot for estimations of biomass and survival. The shoots were cut, washed in distilled water (1 min), placed inside aluminum foil bags, and flash-frozen in liquid nitrogen. Samples were lyophilized in a vacuum chamber at −50 °C for 72 h. The samples were stored in airtight bags in the dark to avoid the accumulation of moisture until their use.

#### 4.2.1. Survival and Biomass Reduction

Twenty-one days after herbicide application, the number of live plants per pot was counted, and the plant shoots collected to determine biomass. Plant shoots were cut, placed in paper bags, and dried for 72 h at 80 °C until reaching constant weight. The percentage of biomass reduction (dry matter) at each dose was obtained by subtracting from the dry matter to untreated plants (0x) and multiplying by 100. Survival was calculated multiplying the number of live plants by 25; survival data were arcsine transformed prior to statistical analysis. Data were analyzed with ANOVA (*p* ≤ 0.05), and when significant differences were found, a Tukey test (*p* ≤ 0.05) was performed to compare among treatments.

#### 4.2.2. Metabolic Fingerprinting Using GC-MS

Ten milligrams of lyophilized tissue was placed in a 2 mL capacity Eppendorf tube, and the tubes were submerged in liquid nitrogen for 2 min. Then, the plant tissue was ground inside the tube with a plastic pestle until obtaining a fine powder. 500 µL of methanol (Mass grade, Fisher Scientific, New Bedford, MA, USA) was added to each tube. To improve metabolite extraction, samples were homogenized for 1 h in a sonifier (Branson 1800, St. Louis, MO, USA). Tubes were centrifuged for 10 min at 13 rpm and the aqueous phase was filtered using a 0.2 µm nylon filter (Millipore, Burlington, MA, USA).

Samples were injected into a gas chromatograph (Clarus 680, Perkin-Elmer Inc., Waltham, MA, USA), equipped with a phase capillary column: 5% diphenyl 95% dimethylpolysiloxane 30 m long, 0.32 mm i.d., 0.25 μm film thickness, temperature limits between −60 a 320/350 °C (Elite-5 MS, Perkin-Elmer Inc., Waltham, MA, USA). The injection was by autosampler. Helium gas was used at a flow rate of 1 mL/min, the flow remained constant, and there was an initial wait time of 0.5 min. The column temperature was initially maintained at 50 °C for 1 min and then ramped to 250 °C at 30 °C/min, remaining at this temperature for a further 10 min. The temperature of the injector was 230 °C. A mass spectrometer (Clarus SQ8T, Perkin-Elmer Inc., Waltham, MA, USA), with an electron impact ionization source (70 eV) in full scan mode was used. The analysis range was 40–500 m/z. The temperatures of the transfer line and ionization source were 230 and 250 °C, respectively.

#### 4.2.3. Data Analyses

Original files of GC-MS were analyzed in the platform XCMS Online [35]. This platform provides the feature detection, retention time correction, peak alignment, and statistical analysis. Since the objective of this study was to obtain a global fingerprint, a non-target metabolomics scope was used. To avoid a wrong interpretation in the name of the metabolites, and for differentiating among metabolites, we only used metabolites with *q*-values ≤ 0.05, and the annotation of each one was according to the *m/z* and the retention time (RT) of each metabolite detected.

The results were represented in an heatmap-bicluster. An ion matrix was constructed using the metabolites with *p*-values ≤ 0.05. The construction of the heatmap was made using the platform Metaboanalyst (www.metaboanalyst.ca) [36]. In this platform, the data were normalized and auto-scaled. The dendrograms used Pearson correlation as a distance function, and the Ward clustering algorithm; the significance of the branches were *p* ≤ 0.05. A supervised learning algorithm (Random forest) was used to measure the importance of each metabolite in the grouping and sample classification. When a metabolite represent an important difference among treatments, the identification of such molecule was made using the NIST library

### 4.3. Experiment 2. Responses of Weeds Treated with Non-Lethal Dose to Posterior Herbicide Application

In order to answer the second question, a second experiment was carried out. The plant material and growth conditions were the same as for experiment 1. In this experiment, the treatments consisted of a first application of herbicides in doses of 0x, 0.0001x, 0.001x and 0.01x. Due to the high mortality observed in treatments with 0.1 and 1x we did not include these treatments in the second experiment. Each treatment had four pots, with six plants per plot.

Twenty-one days after the first herbicide application, the pots were sprayed for a second time using the same dose as the first exposure (0.0001x-T, 0.001x-T and 0.01x-T). At the same time, a set of plants of the same age that had never have been exposed to herbicides were sprayed with herbicides at the same doses (0.0001x-U, 0.001x-U and 0.01x-U). This second application included a control group that was sprayed only with distilled water and adjuvant. Twenty-four hours after the herbicide application, two plants per plot were sampled for metabolomic fingerprinting. The methods for sample collection, processing, injection into GC-MS and data analysis, were the same as described for experiment 1.
